# Efficacy and safety of thread embedding acupuncture for chronic low back pain: a randomized controlled pilot trial

**DOI:** 10.1186/s13063-018-3049-x

**Published:** 2018-12-12

**Authors:** Hyun-Jong Lee, Byung Il Choi, Seungah Jun, Mu Seob Park, Se Jung Oh, Jung Hee Lee, Han Mi Gong, Jae Soo Kim, Young Joon Lee, So-Young Jung, Chang Hyun Han

**Affiliations:** 1 Department of Acupuncture and Moxibustion medicine, College of Korean Medicine, Daegu Haany University, Daegu, 42158 Republic of Korea; 2 Choibyungil 3S Korean medical clinic, Ulsan, 44726 Republic of Korea; 3 Department of Preventive Medicine, College of Korean Medicine, Daegu Haany University, Gyeongsan, 38610 Republic of Korea; 4 Clinical Research Division, Korea Institute of Oriental Medicine, Daejeon, 34054 Republic of Korea

**Keywords:** Thread-embedding acupuncture, Acupuncture, Chronic low back pain

## Abstract

**Background:**

We investigated the efficacy and safety of thread-embedding acupuncture (TEA) for chronic low back pain (LBP) in a randomized controlled pilot trial with the aim of laying the foundation for a large-scale randomized controlled trial on this topic.

**Methods:**

Forty participants were recruited for this two-arm, assessor-blinded randomized controlled pilot trial. The participants were randomly allocated to a TEA group (experimental group) or an acupuncture group (control group). The TEA group received TEA once every 2 weeks for 8 weeks (four sessions in total), while the acupuncture group received acupuncture twice per week for 8 weeks (16 sessions in total). The primary outcome was the visual analog scale (VAS) score for pain and the secondary outcomes were short-form McGill Pain Questionnaire (SF-MPQ) and Oswestry Disability Index (ODI) scores. Assessments were performed at screening and at 2, 4, 6, 8, and 10 weeks after treatment initiation (the 10-week assessment was conducted at 2 weeks after treatment cessation).

**Results:**

Of the 40 participants, 36 completed the study and four dropped out. Both the TEA group and the acupuncture group showed significant improvements in VAS, SF-MPQ, and ODI scores in a time-dependent manner. Furthermore, with regard to ODI, a significant interaction between group and time was observed, with the two groups exhibiting a different pattern of change at 8 weeks according to contrast analysis with Bonferroni’s correction. No serious adverse event occurred, and hematological and biochemical test findings were within normal limits.

**Conclusion:**

This pilot study has provided basic data for a larger clinical trial to demonstrate the efficacy and safety of TEA for chronic LBP.

**Trial registration:**

Clinical Research Information Service of the Korea National Institute of Health, ID: KCT0001819. Registered on 15 February 2016.

## Background

Low back pain (LBP) is a major health problem, with an estimated lifetime prevalence of up to 70–85% [1] and high treatment costs. Most patients with nonspecific acute LBP exhibit an unremarkable natural course. Nonspecific acute LBP generally presents for a brief duration and is self-limited, with an improvement in symptoms within 4 weeks. Subacute LBP is defined as LBP lasting for 4–12 weeks and generally improves with time, albeit in a gradual manner. However, the improvement can be limited and incomplete. A subset of acute LBP patients will develop chronic LBP. The treatment of chronic LBP (lasting for ≥ 12 weeks) is difficult, and the condition does not improve much over time [2]. Approximately 24–80% individuals who experience activity-limiting LBP report a recurrence at 12 months after onset [3]. One year later, approximately 10% patients with acute LBP are either not working or being compensated, and approximately 20% continue to experience clinical symptoms [4]. LBP is associated with substantial morbidity, with individuals reporting that most, if not all, aspects of their lives are significantly affected by chronic pain. Individuals with LBP are often dissatisfied with conventional forms of medical care, including medication, physical therapy, and exercise [5]. Therefore, several patients with chronic LBP are interested in complementary and alternative medicine.

Acupuncture is one of the most frequently used interventions for LBP [6]. A number of meta-analyses of randomized controlled trials evaluating acupuncture for LBP have already been published and have supported the efficacy of this treatment, which has also been proven as an effective supplement to other forms of conventional medical treatments for chronic LBP [7–10].

Thread-embedding acupuncture (TEA) is a new subtype of acupuncture treatment developed from catgut-embedding therapy. In traditional Chinese medicine, catgut-embedding therapy has been used for the treatment of several diseases such as musculoskeletal pain, obesity, chronic urticaria, perimenopausal syndrome, and depressive neurosis [11]. Recently, suture thread was used instead of catgut, which is made from the natural fiber of sheep or goat intestine, in Korea. Although many studies on catgut-embedding therapy have been conducted in China, studies on TEA are lacking; only some case reports have been documented in Korea, and a well-designed clinical study examining the efficacy and safety of TEA is desirable. From this perspective, we designed a randomized controlled pilot trial to evaluate the efficacy and safety of TEA for chronic LBP. The findings of this pilot trial are expected to lay the foundation for a future large-scale randomized controlled trial.

## Methods

### Study design

This two-arm, assessor-blinded randomized controlled pilot trial was conducted at the Daegu Korean Hospital of Daegu Haany University, Daegu, Republic of Korea.

Participants voluntarily participated in the clinical trial and provided written informed consent in accordance with the Declaration of Helsinki after the medical researcher provided sufficient information about the trial. The study was approved by the Institutional Review Board (IRB) of Daegu Oriental Hospital (DHUMC-D-15015-ANS-01) and registered with the Clinical Research Information Service of the Korea National Institute of Health (KCT0001819).

**Fig. 1:**
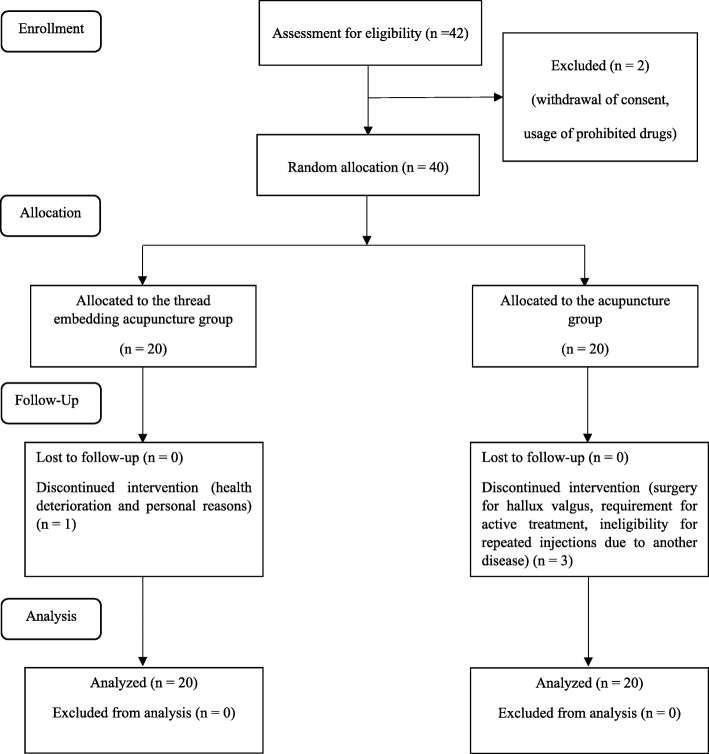
Flow chart for our randomized controlled pilot trial on the efficacy and safety of thread-embedding acupuncture for chronic low back pain

Participants were selected after screening and were randomly assigned to a TEA (experimental) group or an acupuncture (control) group with equal probability. Participants in the TEA group received TEA once every 2 weeks for 8 weeks, while those in the acupuncture group received acupuncture twice per week for 8 weeks (Fig. 1).

Both groups were evaluated using the visual analog scale (VAS), which was the primary outcome measure, and the short-form McGill Pain Questionnaire (SF-MPQ) and Oswestry Disability Index (ODI), which were the secondary outcome measures, at screening and at 2, 4, 6, 8, and 10 weeks after the initiation of treatment.

### Participants

In total, 40 participants with chronic LBP were recruited through advertisements on websites and bulletin boards at the Daegu Korean Hospital of Daegu Haany University.

The inclusion criteria were as follows: lumbar herniated intervertebral disk or spinal stenosis diagnosed on computed tomography or magnetic resonance imaging, LBP for at least 6 months, aged 20–70 years, ability to comprehend or speak in the Korean language, and availability for follow-up during the trial period. Participants were excluded from the study if one or more of the following criteria were fulfilled: adverse event or a hypersensitive reaction caused by acupuncture; cauda equina syndrome; persistently exacerbated symptoms; progressive neurological signs (i.e., sensory or motor changes); previous spinal surgery; neuromuscular scoliosis or neurodegenerative disease; pregnancy, lactation, or plans to conceive; senile dementia; impaired cognitive function or other cerebral disease; severe psychiatric or psychological disorders; refusal to participate in the trial or provide informed consent; alcohol or drug abuse; abnormal findings in renal and/or hepatic function tests; ineligibility considered by the recruiting physician; use of anticoagulant medication; and presence of a pacemaker. Participants were prohibited from taking medication or injections related to LBP.

### Randomization and blinding

Random numbers were generated by a statistician through computerized block randomization using SPSS for Windows (Version 14.0; SPSS Inc., Armonk, NY, USA). Sealed opaque assignment envelopes were used for allocation concealment. The participants were randomly assigned to either the TEA group (*n* = 20) or the acupuncture group (*n* = 20). The outcome assessors and statistician were blinded to randomization and were not involved in the treatment procedures.

### Interventions

#### TEA

**Fig. 2:**
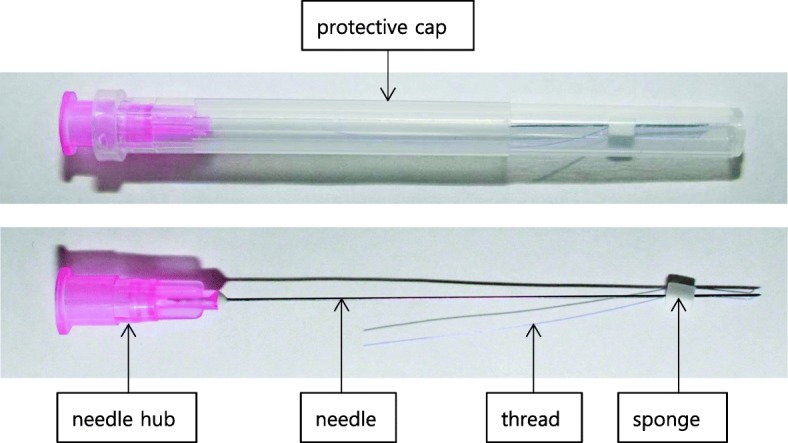
Parts of a thread-embedding acupuncture device

The TEA device (HyunDae MediTech Inc., Wonju, Gangwon, Republic of Korea) was covered with a protective cap before use and comprised four parts: needle hub, needle, thread (polydioxanone suture thread), and sponge (Table 1, Fig. 2). The absorbable thread is a major component of TEA and is internally buried in the treatment areas. The needle gauges and lengths are 27 G × 40 mm and 27 G × 60 mm. The thread length is 50 mm and 90 mm, respectively.

**Table 1:** Function of the different parts of a thread-embedding acupuncture device

Consists	Function
Needle hub	Holds the device at the acupuncture point
Needle	Inserts the absorbable thread into the acupuncture point through the hole of the needle hub
Thread	Absorbable suture thread that is embedded within the body
Sponge	Fixes the needle and absorbable thread
Protective cap	Protects the needle and thread

##### TEA group

**Fig. 3:**
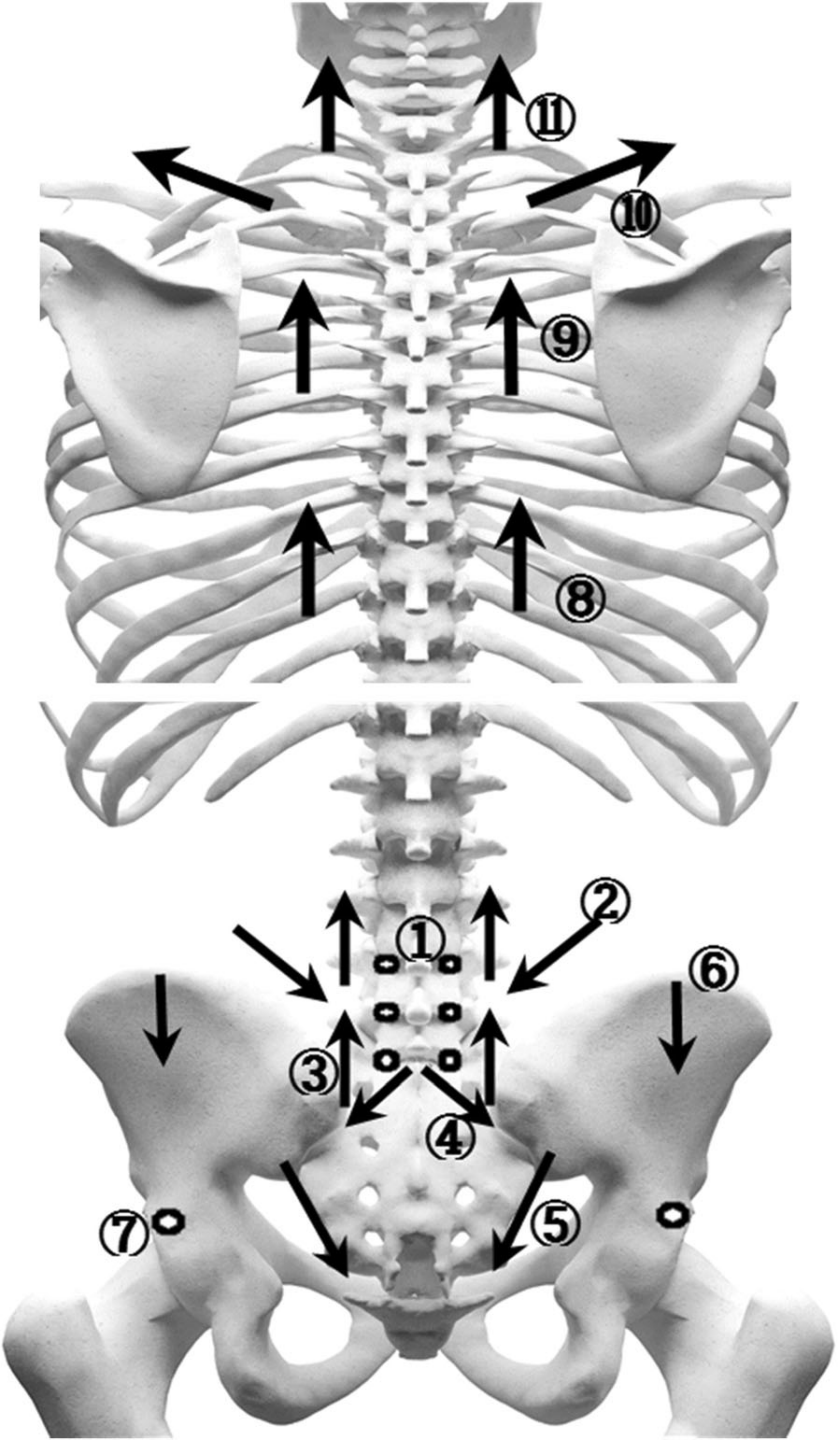
Treatment sites for thread-embedding acupuncture in the dorsal area of the human body

**Fig. 4:**
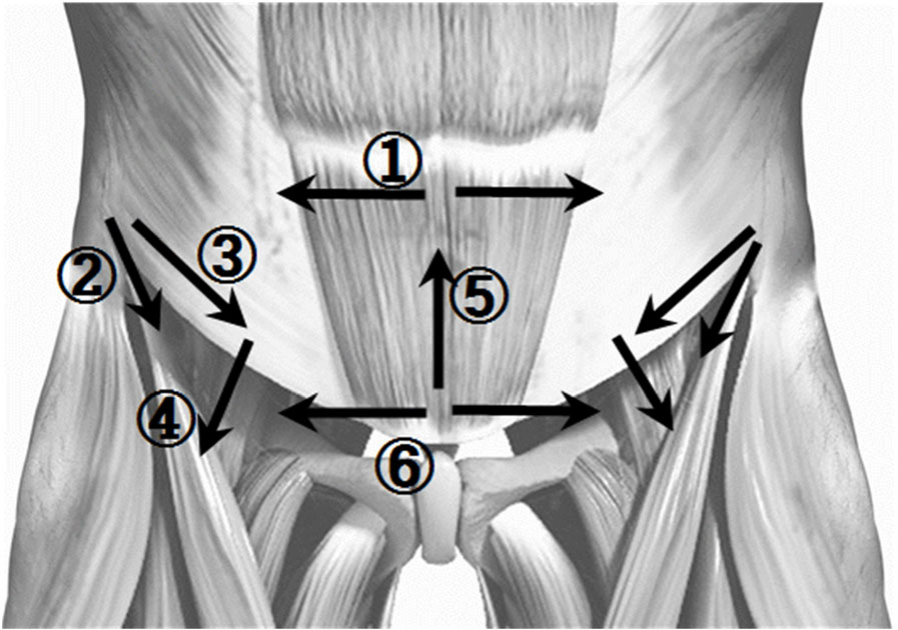
Treatment sites for thread-embedding acupuncture in the abdominal area of the human body

Threads were embedded at 11 sites (28 pieces inserted) in the dorsal area and six sites (11 pieces inserted) in the abdominal area. The 17 treatment sites are described in Tables 2 and 3 and illustrated in Figs. 3 and 4. A Korean medical doctor (KMD) performed the procedure wearing sterilized gloves and a facial mask. First, the KMD marked the treatment sites using a surgical marker and disinfected the sites with a disposable ethanol gauze to prevent infection. During intervention, care was taken to avoid neurovascular damage. TEA was performed once every other week for 8 weeks (four sessions in total).

**Table 2:** Treatment sites for thread-embedding acupuncture in the dorsal area of the human body

	Localization				
	Muscle	Skeleton	Direction of insertion	Needle length	Number of pieces inserted
➀	Intrinsic muscle (spinalis, rotatores)	L3–4, L4–5, L5–S1	Perpendicular insertion	40 mm	6 pieces
➁	Multifidus muscle	L4–5	Oblique insertion	60 mm	2 pieces
➂	Lumbar erector spinae	Sacrum–L5, L3–L1	Transverse insertion	60 mm	4 pieces
➃	Iliolumbar ligament	L5–iliac crest	Oblique insertion	60 mm	2 pieces
➄	Sacroiliac ligament	Posterior inferior iliac spine–coccyx	Oblique insertion	60 mm	2 pieces
➅	Gluteus medius	–	Transverse insertion	40 mm	2 pieces
➆	Piriformis	–	Perpendicular insertion	60 mm	2 pieces
➇	Thoracic vertebrae erector spinae	T7–10	Transverse insertion	60 mm	2 pieces
➈	Thoracic vertebrae erector spinae	T3–5	Transverse insertion	60 mm	2 pieces
➉	Trapezius, levator scapulae	C7	Transverse insertion	60 mm	2 pieces
⑪	Cervical vertebrae erector spinae	C4–7	Transverse insertion	40 mm	2 pieces
Total					28 pieces

**Table 3:** Treatment sites for thread-embedding acupuncture in the abdominal area of the human body

	Localization				
	Muscle	Skeleton	Direction of insertion	Needle length	Number of pieces inserted
➀	Rectus abdominis	–	Transverse insertion	60 mm	2 pieces
➁	Iliacus	Anterior superior iliac spine (ASIS)	Oblique insertion	40 mm	2 pieces
➂	Inguinal ligament	Anterior superior iliac spine (ASIS)	Oblique insertion	60 mm	2 pieces
➃	Iliopsoas	Lesser trochanter of the femur	Transverse insertion	40 mm	2 pieces
➄	Rectus abdominis	–	Transverse insertion	60 mm	1 pieces
➅	Rectus abdominis	Pubis	Transverse insertion	60 mm	2 pieces
Total					11 pieces

##### Acupuncture group

Acupuncture was performed using 0.30 × 40-mm disposable, sterile stainless-steel acupuncture needles (DongBang Acupuncture Inc., Seongnam, Gyeonggi, Republic of Korea). A total of 19 acupuncture points were used, including the unilateral GV3 and bilateral BL23, BL24, BL25, BL26, BL40, BL60, GB30, GB31, and EX-B7. The acupuncture points were localized according to the WHO Standard Acupuncture Point Location in the Western Pacific Region [12].

Following skin disinfection, the acupuncture needles were inserted perpendicular to a depth of 5 to 20 mm. Subsequently, the KMD connected the crocodile clips of a PG 306 electronic stimulator (Suzuki Iryoki Co., Ltd., Tokyo, Japan) to the handles of the acupuncture needles at the bilateral BL23 and BL25 points. A continuous wave at 4 Hz was delivered to the acupuncture needles for 20 ± 5 min per session. Acupuncture was performed twice per week for 8 weeks (16 sessions in total).

### Outcome measures

#### Primary outcome measure

VAS is an instrument for the self-assessment of pain and comprises a scale with 0 cm and 10 cm at either end of a straight line. The participants marked a point on the line to indicate their level of average pain in the recent 2 weeks, and the VAS score was determined by measuring the distance from the left end of the line to the marked point. A score of 0 indicated no pain and a score of 10 indicated severe pain [13].

#### Secondary outcome measures

##### SF-MPQ

The SF-MPQ is an abbreviated version of the widely used MPQ and is a combination of the VAS, a descriptive scale, and a Present Pain Intensity (PPI) scale. The descriptive scale is based on 15 selected words, including 11 in the sensory domain and four in the emotional domain. Each item is scored from 0 to 3, with 0 indicating no symptoms, 1 indicating mild symptoms, 2 indicating moderate symptoms, and 3 indicating severe symptoms [14]. The total score is obtained by summating the individual scores for all 15 items, with higher scores indicating more severe LBP. The PPI scale measures the current pain severity on six levels, with scores of 0, 1, 2, 3, 4, and 5 indicating no pain, mild pain, discomforting pain, distressing pain, horrible pain, and excruciating pain, respectively [15].

##### ODI

The ODI [16] is used to measure LBP-related dysfunction and includes 10 questions pertaining to daily activities, including pain intensity, personal care, lifting, walking, sitting, standing, sleeping, sexual life, social life, and travelling. Each question is scored from 0 to 5, with lower scores indicating lesser disability. We used the validated Korean version of the ODI [17] in the present study.

To obtain the ODI score, each of the points awarded to the chosen answers are summated and the overall score ranges between 0 and 50. If a section about sex life is not applicable, the total possible score was calculated as 45 not 50. In order to express as a percentage, the index is calculated by dividing the summated score by the total possible score, which is then multiplied by 100.

#### Adverse events and safety

All expected or unexpected adverse events related to TEA or acupuncture were reported to the researcher by the participants or observed by the researcher. All participants who received TEA or acupuncture for at least one session underwent an adverse event evaluation. Specifically, hematological and biochemical tests were performed to evaluate the safety of TEA or acupuncture at screening and at week 8. The measures included the red blood cell (RBC) count; total white blood cell (WBC) count; differential WBC count; hemoglobin level; hematocrit; platelet count; erythrocyte sedimentation rate (ESR); and aspartate aminotransferase (AST), alanine aminotransferase (ALT), blood urea nitrogen (BUN), creatinine, serum sodium, serum potassium, and serum chloride levels.

The TEA group participants additionally underwent hematological and biochemical tests at 2 days after the first treatment session in order to rule out infection, which is the largest complication of TEA.

#### Sample size calculation and statistical analysis

As this is a pilot trial, each group included 20 participants on the basis of practical considerations. A total of 40 participants were included, which is larger than the minimum number recommended for pilot studies [18].

All statistical analyses were based on the Statistics Guidelines for Clinical Trials [19] and were conducted using SPSS 19.0 for Windows (Version 14.0; SPSS Inc., Armonk, NY, USA). A *p* value of < 0.05 was considered statistically significant. The last observation carried forward method was used for missing data from drop-outs. To assess baseline demographic and clinical characteristics between groups, Student’s *t* test for parametric analysis or the Mann-Whitney *U* test for nonparametric analysis was used. Chi-square (*χ*^2^) tests were used for the analysis of qualitative data.

Repeated-measures analysis of variance (ANOVA) was conducted to assess between-group differences with regard to changes in VAS, SF-MPQ, and ODI. If a significant interaction between group and time was observed, the time at which the two groups exhibited differences in the pattern of change was examined by contrast analysis with Bonferroni’s correction.

## Results

### Baseline characteristics

The baseline characteristics and outcome measurements for the 40 participants allocated to the two groups are shown in Table 4. There was no significant difference in any parameter between the two groups.

**Table 4:** Baseline characteristics and outcome measurements for participants with chronic low back pain who received TEA or acupuncture

Variable	Group		*p* value
	TEA group (*n* = 20)	Acupuncture group (*n* = 20)	
Sex, *n* (%)			
Male	3 (15.0)	5 (25.0)	0.429^a^
Female	17(85.0)	15(75.0)	
Mean age, years	50.35 ± 11.45	51.9 ± 8.99	0.871^b^
VAS	6.04 ± 0.29	5.91 ± 0.34	0.655^b^
SF-MPQ			
Descriptive scale	18.85 ± 1.23	19.40 ± 1.65	0.791^c^
PPI	2.65 ± 0.15	2.45 ± 0.15	0.273^b^
ODI	35.20 ± 1.93	41.56 ± 2.88	0.075^c^

Values are expressed as means ± standard deviations

*ODI*, Oswestry Disability Index, *PPI* Present Pain Intensity scale, *SF-MPQ* short-form McGill Pain Questionnaire, *TEA* thread-embedding acupuncture, *VAS* visual analog scale

^a^Chi-square (*χ*^2^) test

^b^Mann-Whitney *U* test

^c^Student’s *t* test

### Enrollment and drop-out rates

A total of 42 participants were screened to enroll 40 participants in the trial (acceptance rate of 95.2%). One of the 42 eligible participants withdrew consent, while another consumed prohibited drugs. In total, 36 participants completed the trial and four dropped out, including one from the TEA group and three from the acupuncture group. The TEA group participant withdrew because of health deterioration and personal reasons, while the acupuncture group participants withdrew because of admission to another hospital for hallux valgus surgery, the requirement for active treatment of severe LBP after cleaning the bathroom, and ineligibility for repeated injections due to another disease, respectively. The compliance rate for the remaining 36 participants was 100%.

### Primary outcome

**Fig. 5:**
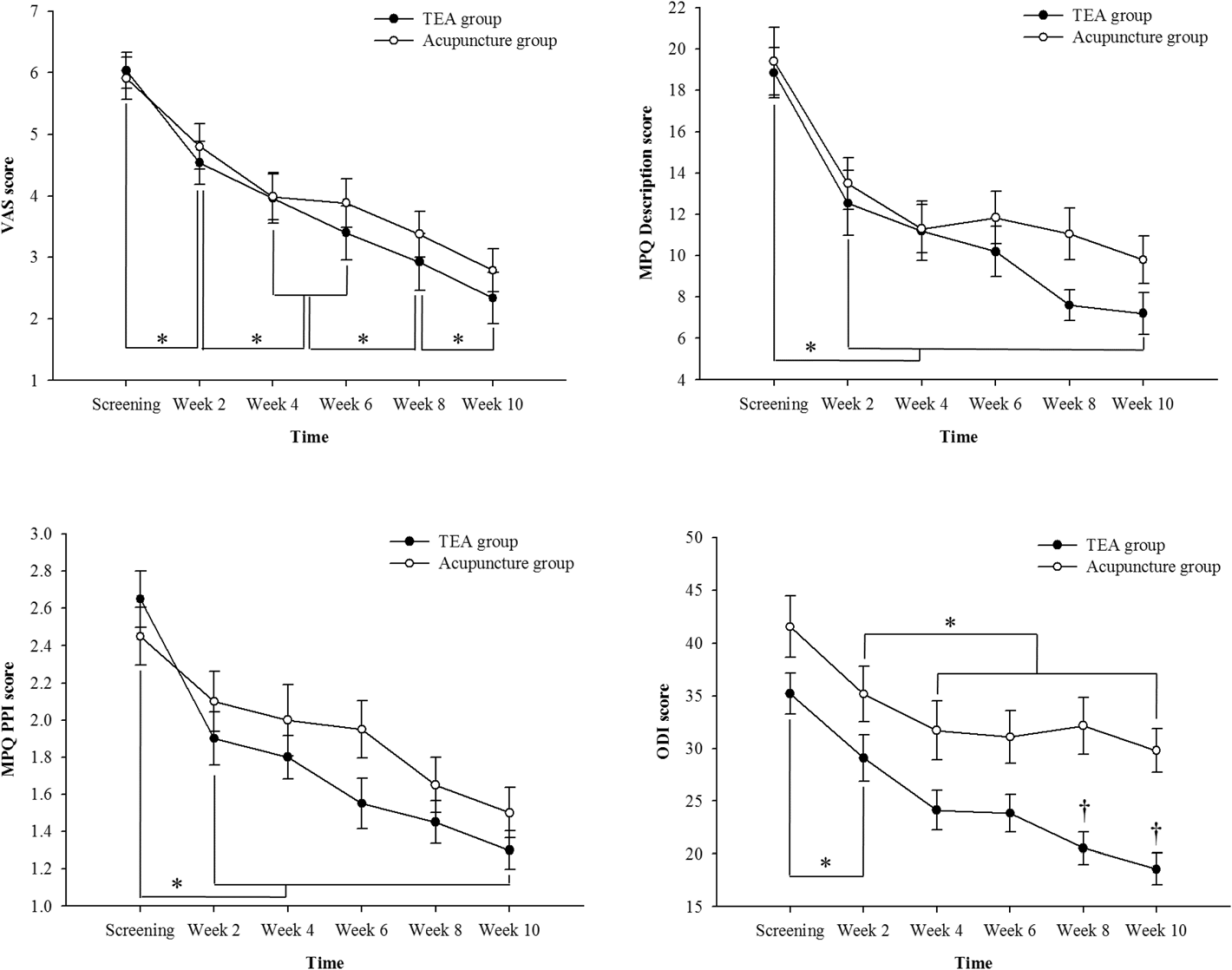
Changes in VAS, SF-MPQ, and ODI scores in TEA and acupuncture groups. ^*^ *p* < 0.05: statistically significant difference according to repeated-measures analysis of variance (ANOVA) by contrast analysis with Bonferroni’s correction on time. ^†^ *p* < 0.05: statistically significant difference according to repeated-measures analysis of variance (ANOVA) by contrast analysis with Bonferroni’s correction on time and group interaction. *MPQ* short-form McGill Pain, *ODI* Oswestry Disability Index, *PPI* Present Pain Intensity scale, *TEA* thread-embedding acupuncture, *VAS* visual analog scale

According to repeated-measures ANOVA (Table 5 and Fig. 5), both groups showed a significant improvement in VAS scores in a time-dependent manner. Although the TEA group tended to exhibit a greater improvement compared with the acupuncture group, there were no significant differences between groups. Repeated-measures ANOVA also found no significant differences between the two groups. There was no interaction between time and group.

**Table 5:** Changes in VAS, SF-MPQ, and ODI scores in participants with chronic low back pain who received TEA or acupuncture

Variable	Group	Time, mean(se)						*p* value		
		Screening^a)^	2 week^b)^	4 week^c)^	6 week^d)^	8 week^e)^	10 week^f)^	time	group	time × group
VAS	TEA group	6.04 ± 0.29	4.54 ± 0.35	3.97 ± 0.41	3.40 ± 0.44	2.93 ± 0.47	2.34 ± 0.42	< 0.001^c^ (a > b > c,d > e > f)^d^	0.556	0.749
	Acupuncture group	5.91 ± 0.34	4.81 ± 0.37	3.99 ± 0.37	3.89 ± 0.40	3.38 ± 0.38	2.79 ± 0.35			
SF-MPQ										
Descriptive scale	TEA group	18.85 ± 1.23	12.55 ± 1.58	11.20 ± 1.45	10.20 ± 1.25	7.60 ± 0.75	7.20 ± 1.02	< 0.001^c^ (a > b,c,d,e,f)^d^	0.248	0.355
	Acupuncture group	19.40 ± 1.65	13.50 ± 1.24	11.30 ± 1.18	11.85 ± 1.28	11.05 ± 1.25	9.80 ± 1.16			
PPI	TEA group	2.65 ± 0.15	1.90 ± 0.14	1.80 ± 0.12	1.55 ± 0.14	1.45 ± 0.11	1.30 ± 0.11	< 0.001^c^ (a > b,c,d,e,f)^d^	0.242	0.389
	Acupuncture group	2.45 ± 0.15	2.10 ± 0.16	2.00 ± 0.19	1.95 ± 0.15	1.65 ± 0.15	1.50 ± 0.14			
ODI	TEA group	35.20 ± 1.93	29.09 ± 2.19	24.14 ± 1.88	23.84 ± 1.80	20.54 ± 1.57	18.56 ± 1.51	< 0.001^c^ (a > b > c,d,e,f)^d^	0.004^b^	0.038^a^ (a,b,c,d > e,f)^d^
	Acupuncture group	41.56 ± 2.88	35.17 ± 2.64	31.70 ± 2.79	31.09 ± 2.52	32.15 ± 2.72	29.80 ± 2.09			

Values are expressed as means ± standard deviations

*TEA* thread-embedding acupuncture, *VAS* visual analog scale, *SF-MPQ* short-form McGill Pain Questionnaire, *PPI* Present Pain Intensity scale, *ODI* Oswestry Disability Index

^a^*p* < 0.05, ^b^*p* < 0.01, ^c^*p* < 0.001: statistically significant difference according to repeated-measures analysis of variance (ANOVA)

^d^*p* < 0.05: statistically significant difference according to multiple comparisons by contrast analysis with Bonferroni’s correction

### Secondary outcomes

According to repeated-measures ANOVA (Table 5 and Fig. 5), both groups showed a significant improvement in SF-MPQ and ODI scores in a time-dependent manner. Although the TEA group tended to exhibit a greater improvement in SF-MPQ compared with the acupuncture group, repeated-measures ANOVA showed no significant differences between groups. However, a significant interaction between group and time was observed with regard to the ODI, with the two groups exhibiting a different pattern of change at 8 weeks by contrast analysis with Bonferroni’s correction.

### Adverse events and safety

No serious adverse events were recorded. Two participants in the acupuncture group experienced itching in the low back area, which was considered to be associated with the treatment. Hematological and biochemical test findings at screening and at week 8 were within normal limits. In particular, hematological and biochemical tests performed at 2 days after the first TEA session showed no infection in any participant.

## Discussion

TEA is a new acupuncture technique that involves the use of a specially designed device for the insertion of absorbable thread (extraneous substance) at an acupoint to provide continuous stimulation. In the early days in Korea, TEA was commonly used for cosmetic applications and obesity treatment [20]. Recently, KMDs have started using TEA for the treatment of various musculoskeletal diseases. There are many reports on TEA for musculoskeletal pain, including ankle sprain, patellar dislocation, and lumbar disk herniation [21–23]. However, most of these reports described single cases, and there is no randomized controlled study to date.

In the present study, we evaluated the efficacy and safety of TEA for chronic LBP and found significant improvements in VAS, SF-MPQ, and ODI scores in a time-dependent manner in both TEA and acupuncture groups. These findings suggest that both TEA and acupuncture may be effective for pain control. Furthermore, the ODI was associated with a significant interaction between group and time, with the two groups exhibiting a different pattern of change at 8 weeks according to contrast analysis with Bonferroni’s correction. Considering that ODI is the most standard measurement for low-back-specific dysfunction, our findings suggest that TEA maybe more effective in improving the disability of patients compared with conventional acupuncture. Also more than 8 weeks of TEA treatment is required to achieve effects better than those of acupuncture.

Even if a patient with chronic LBP undergoes active treatment, improvement is very slow. In addition, patients may be reluctant to accept acupuncture treatment because it requires frequent visits to the doctor. TEA is performed once every 2 weeks and may provide somewhat better effects compared with conventional acupuncture treatment. If TEA can provide better effects with fewer visits compared with conventional acupuncture, it can also decrease the overall cost of treatment and save time.

Not many studies have investigated the mechanism of action of TEA, which is considered to act through both the acupuncture and the suture thread. The insertion process is similar for both TEA and acupuncture; however, in TEA, the suture thread remains inside the body for continuous biostimulation. Recently, it was reported that the embedded thread, which dissolves by slow hydrolysis in the tissue, may play a role as a long-lasting stimulator of collagen fiber formation through the downregulation of c-Jun *N*-terminal kinase (JNK) and matrix metalloproteinase (MMP)-9 activity [24].

We assumed that TEA can provide significant amelioration of chronic LBP by continuous stimulation of the core muscles and ligaments around the spine and abdomen. The core muscles play an important role in stabilizing the spine and are used in exercise therapy for chronic LBP. If chronic LBP persists for a long time, the muscles around the cervical and thoracic spine can also become weak. Accordingly, the erector spinae, trapezius, and levator scapulae should also be treated with muscles in the lumbar region. Therefore, we performed TEA using the core, back, and neck muscles, which resulted in similar effects with lesser visits compared with conventional acupuncture. The precise mutual relationship between the thread and surrounding tissues remains unclear because of the lack of previous studies, and further studies should evaluate the mechanism of action of TEA in more detail.

Although TEA is frequently used in clinics and hospitals for Korean medicine, there is lack of evidence regarding its safety. TEA is generally known to be safe. However, because it is more invasive than acupuncture and involves the insertion of a thread, the possibility of infection must be considered. In the present study, we tested for infection by performing hematological and biochemical tests at screening and 8 weeks after treatment initiation in both groups and at 2 days after the first treatment session in the TEA group. The findings at all time points were within normal limits, supporting the safety of TEA. Recently, tender, erythematous, subcutaneous nodules at the point where the catgut was embedded were reported and considered to be a foreign-body reaction [25]. Thread used in TEA is polydioxanone and has already been widely used as a suture thread [26]. Therefore, TEA seems safer than catgut-embedding therapy in the foreign-body reaction. Although two participants from the acupuncture group in the present study complained of itching on the low back, no serious adverse effects or skin problems were recorded for any participants. Therefore, our results suggest that TEA may be a safe procedure in 10 weeks. However, the long-term adverse events of TEA should be examined.

The present study has some limitations. Even though TEA exhibited similar results in a fewer number of visits compared with acupuncture, the precise underlying mechanism is not clear and needs to be clarified through additional research. Participants and practitioner had not been blinded in this study. Especially in the TEA study, it is difficult to develop a sham device for the purpose of blinding. First of all, a participant-blinded clinical trial is necessary for further study. Blinding participants by performing TEA without a thread can be an alternative. Inserting the needle after removing the thread is considered to produce participant blindness and evaluate the effect of the thread. However, this method cannot also blind practitioners and, therefore, cannot avoid high risk of performance bias. In this 10-week study, no adverse effects of TEA have occurred. However, we cannot be convinced that TEA is totally safe. TEA is still invasive and dangerous in terms of inserting threads. Further studies are needed on infection, hematological examination, foreign-body reaction to the thread, and neurovascular injury. In addition, factors that can affect results, such as participant’s expectation to the study participation or the allocated interventions, socioeconomic status and disease duration, were not measured. It is advisable to consider unmeasured confounders in a further larger-scale study. Also, as the present study is a pilot study, further large-scale studies are needed to demonstrate the exact efficacy and safety of TEA.

## Conclusions

This pilot study suggested the possibility that treating chronic LBP using TEA may be safe and effective in the short-term. This result has provided basic data for a larger clinical trial on TEA for chronic LBP.

## Abbreviations

ANOVA: Analysis of variance
IRB: Institutional Review Board
KMD: Korean medical doctor
ODI: Oswestry Disability Index
PPI: Present Pain Intensity
SF-MPQ: Short-form McGill Pain Questionnaire
TEA: Thread-embedding acupuncture
VAS: Visual analog scale
